# Thoracic Kyphosis on Chest CT Scans Is Associated With Incident Vertebral Fractures in Smokers

**DOI:** 10.1002/jbmr.3672

**Published:** 2019-02-27

**Authors:** Mayke J van Dort, Johanna HM Driessen, Elisabeth APM Romme, Piet Geusens, Paul C Willems, Frank WJM Smeenk, Emiel FM Wouters, Joop PW van den Bergh

**Affiliations:** ^1^ NUTRIM School of Nutrition and Translational Research in Metabolism Maastricht University Medical Centre+ (MUMC+) Maastricht the Netherlands; ^2^ CAPHRI Care and Public Health Research Institute Maastricht University Medical Centre+ (MUMC+) Maastricht the Netherlands; ^3^ Department of Clinical Pharmacy and Toxicology Maastricht University Medical Centre+ (MUMC+) Maastricht the Netherlands; ^4^ Department of Respiratory Medicine Catharina Hospital Eindhoven the Netherlands; ^5^ Department of Internal Medicine, Rheumatology Maastricht University Medical Centre+ (MUMC+) Maastricht the Netherlands; ^6^ Department of Orthopedic Surgery Maastricht University Medical Center + (MUMC+) Maastricht the Netherlands; ^7^ School of Health Professions Education Faculty of Health Medicine and Life Sciences Maastricht University Medical Centre+ (MUMC+) Maastricht the Netherlands; ^8^ Department of Respiratory Diseases Maastricht University Medical Centre+ (MUMC+) Maastricht the Netherlands; ^9^ Department of Internal Medicine VieCuri Medical Centre Venlo the Netherlands

**Keywords:** FRACTURE RISK ASSESSMENT, BIOMECHANICS, SCREENING, KYPHOSIS ANGLE, SMOKERS

## Abstract

Greater kyphosis angles lead to increased loading on vertebral bodies in computational models. However, results about the relationship between severity of kyphosis and incident vertebral fracture (VF) risk have been conflicting. Therefore, the aim of this study was to evaluate associations between 1) prevalent VFs and severity of kyphosis, and 2) severity of kyphosis and incident VF risk in smokers with or without chronic obstructive pulmonary disease (COPD). Former and current smokers with or without COPD were included. CT scans were made at baseline, 1‐year, and 3‐year follow‐up. VFs were evaluated on superposed sagittal CT reconstructions. Kyphosis was measured as the angle between the lines above T_4_ and below T_9_ or T_12_. We included 1239 subjects (mean age 61.3 ± 8.0 years, 61.1% male, 80.6% with COPD), of whom 253 (20.4%) had a prevalent VF and 294 (23.7%) an incident VF within 3 years. Presence, number, and severity of prevalent VFs were associated with a greater kyphosis angle. The mean increase in kyphosis angle within 3 years was small but significantly greater in subjects with incident VFs compared with those without (2.2 ± 4.1 versus 1.2 ± 3.9 degrees, respectively, for T_4_ to T_12_ angle, *p* < 0.001). After adjustment for bone attenuation (BA) and prevalent VFs, baseline kyphosis angle was associated with incident VFs within 1 and 3 years (angle T_4_ to T_12_ per +1 SD, hazard ratio [HR] = 1.34 [1.12–1.61] and HR 1.29 [1.15–1.45], respectively). Our data showed that a greater kyphosis angle at baseline was independently associated with increased risk of incident VFs within 1 and 3 years, supporting the theory that greater kyphosis angle contributes to higher biomechanical loads in the spine. © 2019 American Society for Bone and Mineral Research.

## Introduction

Chronic obstructive pulmonary disease (COPD) is caused by significant exposure to noxious particles and gases, most often tobacco smoking but also exposure to air pollution.^1^, ^2^, ^3^, ^4^ It is characterized by chronic airflow limitation that is caused by a mixture of small airways disease (eg, obstructive bronchiolitis) and parenchymal destruction (emphysema). Although COPD is primarily a pulmonary disease, there are significant comorbidities and extrapulmonary effects, such as cardiovascular disease, diabetes, muscle wasting, and osteoporosis.^5^, ^6^, ^7^, ^8^ The reported prevalence of vertebral fractures (VFs) is high among patients with COPD (9% to 79%),^9^, ^10^, ^11^, ^12^, ^13^, ^14^, ^15^, ^16^, ^17^, ^18^ and we have recently shown that incident VF risk is high in COPD patients and (former) smokers without COPD with one or more prevalent VFs.^19^

Hyperkyphosis, an excessive increase in thoracic spinal curvature, is a common condition estimated to affect 20% to 40% of the older population.^20^ However, because normal kyphosis is increasing with age, cut‐off values defining hyperkyphosis are lacking.^21^ Although presence of VFs is often reported to be the main cause of increased kyphosis, more than half of the hyperkyphotic patients do not have VFs.^21^ Other possible causes can be Scheuermann's disease, intervertebral disk degeneration, and muscle weakness.^21^

Consequences of increased kyphosis are decreased gait performance,^22^ increased fall‐risk tendency,^23^ and decreased quality of life.^22^ Although evidence is limited, it is a common belief that increased thoracic kyphosis limits pulmonary capacity.^24^ VFs are associated with increased kyphosis, and additionally, increased kyphosis can contribute to increased fracture risk, even when adjusted for prior fracture history.^25^, ^26^

A computational model showed that during most daily activities, loading is highest in the thoracolumbar and lumbar spine.^27^ In addition, increase in thoracic kyphosis was associated with increased loading mainly in the thoracolumbar spine, suggesting that a greater kyphosis angle is related to increased VF risk.^28^

However, clinical data on the relationship between increased kyphosis and incident VF risk have been conflicting. Roux and colleagues assessed 1624 subjects from the Spinal Osteoporosis Therapeutic Intervention (SOTI) and Treatment of Peripheral Osteoporosis (TROPOS) studies, and found relative risks (RRs) of 1.30 (1.00–1.68) and 1.42 (1.08–1.86) when the highest T_4_ to T_12_ angle tertile was compared with the medium and to the lowest tertile, respectively, after adjustment for age, body mass index (BMI), spine bone mineral density (BMD), and prevalent VFs.^29^ In contrast, Katzman and colleagues assessed 3038 women with low BMD from the Fracture Intervention Trial and did not find a significant influence of increased C_7_ to T_12_ kyphosis angle on incident VF risk after adjustment for prevalent VFs.^30^

Because smokers with or without COPD are at increased risk of VFs and chest CT scans are regularly made especially in COPD patients, it would be interesting to know whether thoracic kyphosis as measured on CT is an independent risk factor for incident VFs. Therefore, our aim was to evaluate the associations between 1) prevalent VFs and thoracic kyphosis angle, and 2) between thoracic kyphosis angle and incident VFs in current and former smokers with or without COPD.

## Materials and Methods

### Subjects

Current and former smokers with or without COPD from the ECLIPSE study (Evaluation of COPD Longitudinally to Identify Predictive Surrogate Endpoints; http://Clinicaltrials.gov identifier NCT00292552; GlaxoSmithKline study SCO104960) were included. The ECLIPSE study is a noninterventional, observational, multicenter study that was started to search underlying mechanisms of disease progression in subjects with COPD and to identify biomarkers that may serve as surrogate endpoints and therefore could measure disease progression. Detailed inclusion and exclusion criteria were described elsewhere.^(31–33)^ In short, subjects aged 40 to 75 years, with a smoking history of at least 10 pack‐years (1 pack‐year = 20 cigarettes [1 pack] per day for 1 year), either with moderate (stage II) to very severe (stage IV) COPD (stage II: 50% ≤ FEV_1_ <80% predicted [FEV_1_ = forced expiratory volume in 1 second], and FEV_1_/FVC <0.70 [FVC = forced vital capacity]; stage III: 30% ≤ FEV_1_ <50% predicted, FEV_1_/FVC <0.70; stage IV: FEV_1_ < 30% predicted, FEV_1_/FVC <0.70) according to the GOLD guidelines (Global Initiative for Chronic Obstructive Lung Disease) or without COPD (FEV_1_ >85% predicted, FEV_1_/FVC >0.70) were included. Both current and former smokers were eligible. Subjects with respiratory diseases other than COPD were excluded, as well as subjects with an exacerbation requiring treatment in the 4 weeks before enrollment, and subjects using oral glucocorticosteroids (GC) at baseline. Only subjects with complete set of CT scans at baseline, 1‐year, and 3‐year follow‐up were included; subjects with scans of insufficient quality or lack of clear anatomic landmarks to identify vertebrae were not eligible for this study.^19^

### Measurements

Demographic and pulmonary parameters were collected at baseline, 1‐year, and 3‐year follow‐up. Also, pack‐years and smoking status (current or former) were evaluated. Detailed information can be found elsewhere.^31^, ^32^, ^33^

#### CT scan analyses and VF assessment

At baseline, 1‐year, and 3‐year follow‐up, CT scans of the chest were performed at full inspiration (120 kV peak, 40 mAs, 1.00 or 1.25‐mm volumetric acquisition, General Electric [GE] or Siemens). Of all sagittal reformats containing the spine, the contrast was adjusted to (partly) eliminate soft tissue. Subsequently, all sagittal reformats containing the spine were superposed to create simulated lateral X‐ray 2D images using Matlab (version R2013a, MathWorks, Natick, MA, USA). Images were exported in DICOM‐format.^19^, ^34^

VF assessment was described in detail elsewhere.^19^ In short, vertebrae were first visually evaluated and were, after exclusion of deformities due to Scheuermann's disease, Schmorl's noduli, or platyspondyly, marked as “VF” or “no VF.” Next, in case of positive evaluation, vertebrae were morphometrically assessed using SpineAnalyzer software (Optasia Medical, Cheadle, UK).^34^, ^35^, ^36^ Based on the amount and location of height loss as measured by the software, VFs were classified according to the scoring method proposed by Genant and colleagues as grade 1 (mild: 20% to 25% height reduction in vertebral body), grade 2 (moderate: 25% to 40%), or grade 3 (severe: >40% height loss in vertebral body).^37^ In addition, severity and number of VFs from T_4_ to L_1_ was expressed as the spinal deformity index (SDI),^38^ which is calculated as the sum of the grades of all VFs within the subject (eg, a subject with two grade 2 VFs and one grade 3 VF has an SDI of 7).

If one or more VFs (any shape or grade) were quantitatively assessed on the 3‐year scan, the 1‐year scan was also quantitatively assessed. If VFs were quantitatively assessed on the 1‐year scan, the baseline scan was also assessed. Incident VFs were defined as new VFs (from no VF to any grade of VF) or worsening VFs (eg, from a grade 2 to a grade 3 VF) between baseline and 1‐year follow‐up, and between baseline and 3‐year follow‐up.

According to Genant and colleagues, a VF can be wedge shaped (anterior height loss), biconcave shaped (middle height loss), or crush shaped (height loss of total vertebral body). SpineAnalyzer morphometry software uses the following guidelines to classify shapes of the VFs:

$$Deformity\;wedge\;\%=100*\left(1-h_{A}/h_{P}\right)$$

$$Deformity\;biconcave\;\%=100*\left(1-h_{M}/h_{P}\right)$$

$$Deformity\;crush\;\%=100*\left(1-\min(\max(h_{Pi}/h_{Pi-1},h_{Ai}/h_{Ai-1}\right),\max\left(h_{Pi}/h_{Pi+1},h_{Ai}/h_{Ai+1}\right)$$

‐ h_A_ = anterior height of vertebral body
‐ h_P_ = posterior height of vertebral body
‐ h_M_ = mid height of vertebral body
‐ i = level of vertebra measured
‐ i +1 or I −1 = vertebral level above resp. below the measured vertebra

If both posterior and mid height of the vertebral body showed height loss (for example, a VF with 41% mid height loss and 24% anterior height loss), SpineAnalyzer indicated both VF shapes (biconcave and wedge). In such cases, VFs were scored according to the largest deformation.

#### Bone attenuation

Bone attenuation (BA) was measured on CT in vertebrae T_4_ to T_12_, using a self‐written algorithm in Matlab (R2013a, MathWorks). BA was measured semiautomatically in cubic areas of approximately 275 mm^3^ each (slightly varying due to voxel size). Vertebrae that were diagnosed with a VF or that showed other abnormalities such as Scheuermann's disease, Schmorl's noduli, or platyspondyly (in concertation of MvD, PG, and JvdB) were excluded from BA measurement. BA was measured as the mean of T_4_ to T_12_ and expressed in Hounsfield Units (HU).

#### Kyphosis

**Figure 1:**
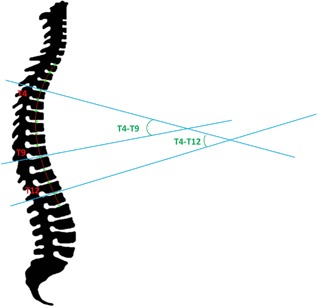
Measurement of kyphosis angles (T_4_ to T_9_ and T_4_ to T_12_) by third‐order polynomial. User‐indicated points (green) were placed centered in the intervertebral disks, and a third‐order polynomial (red) was fit through these points. The angle between T_4_ and T_9_ was measured as the angle between two lines (blue), above T_4_ and below T_9_, perpendicular to the third‐order polynomial closest to the user‐indicated points above T_4_ and below T_9_, respectively. The lines above T_4_ and below T_12_ were used to measure the angle between vertebrae T_4_ and T_12_.

To measure kyphosis angles, a third‐order polynomial was fit through the spine based on user‐indicated points centered in the intervertebral disks (Fig. 1, self‐written algorithm in Matlab). The third‐order polynomial was fitted in the sagittal (2D) plane; therefore, curvature in the coronal plane did not influence the polynomial. Large curvature in the coronal plane, such as observed in scoliotic patients, resulted in unclear images of the vertebrae on the simulated X‐ray images, and therefore these patients were not included in this study. Kyphosis was measured as the angle between two lines perpendicular to the polynomial, crossing the polynomial closest to the user‐indicated points in the intervertebral disks. The angles between T_4_ and T_9_ (lines crossing polynomial in the intervertebral disks above T_4_ and below T_9_) and between T_4_ and T_12_ (lines crossing above T_4_ and below T_12_) were measured (Fig. 1). The mean *r* ^2^ for the degree of fit of the polynomial to the user‐indicated points was 0.99 (range 0.9323 to 0.9998), and the intraclass correlation coefficient (ICC) of triple measurements of a subset of *n* = 25 scans was excellent (ICC > 0.95, data not shown). In addition, kyphosis angles measured using this method were compared with kyphosis angles between vertebral endplates measured using Surgimap software (Surgimap, Nemaris Inc., New York, NY, USA; available via http://www.surgimap.com) and showed very good correlations (*n* = 92 and *n* = 77 for T_4_ to T_9_ and T_4_ to T_12_ angles, respectively; *r* ^2^ > 0.85, data not shown) for both the T_4_ to T_9_ and the T_4_ to T_12_ angle.

### Outcome measures and statistics

Main outcome measures were baseline kyphosis angles, change in kyphosis angles, and incidence of VFs.

Possible confounders were age, sex, presence of COPD, BMI, pack‐years, smoking status (current or former), BA, and prevalent VFs. Age, sex, and presence of COPD were included in all models; other confounders were included if they influenced the beta‐coefficient of the main exposure more than 5% or when consensus consisted within the team of researchers supported by evidence from literature.

To evaluate associations between prevalent VFs and kyphosis angle and between incident VFs and change in kyphosis angle, linear regression models were used (SAS 9.3, SAS Institute, Cary, NC, USA; REG procedure).

Because the prevalence of the outcome measure “incident VFs” is 10% over a 1‐year time period and 24% over the 3‐year time period, Cox proportional hazard models (PHREG procedure) were used to evaluate the association between baseline kyphosis and incident VFs. Hazard ratios (HRs) are given with 95% confidence interval (95% CI), and are given per standard deviation for continuous variables.

## Results

Of a total of 2298 ECLIPSE subjects (327 subjects without and 1971 with COPD), 1478 subjects had the complete set of CT scans (baseline, 1‐year, and 3‐year follow‐up). Of these, 237 subjects were excluded because of various reasons, including scan quality (noise, missing slices, incorrect slice spacing; *n* = 156); anatomy (could not identify T_1_/vertebral levels, deformation of the spine; *n* = 14); failure of the method to edit CT scans (slice numbers not in ascending order and/or not starting at 0 or 1, problems with white balance in Matlab, or unclear adapted CT images; *n* = 60); or use of oral GC at baseline (*n* = 7). Additionally, 2 subjects were excluded because of multiple deformations other than VFs (platyspondyly, Scheuermann's disease; for flowchart and characteristics of included and excluded subjects, see van Dort and colleagues^19^). Thus, 1239 subjects were included (999 subjects with and 240 subjects without COPD). Baseline characteristics are given in Table 1. There were 133 (11%) subjects using inhaled steroids at baseline, 123 (10%) at 1‐year and 116 (9%) at 3‐year follow‐up. There were 23 (2%) subjects using oral steroids at the time of 1‐year follow‐up and 47 (4%) at 3‐year follow‐up.

**Table 1:** Baseline Characteristics

	Included subjects		Men		Women	
	*n* = 1239		*n* = 757		*n* = 482	
Age (years: mean, SD)	61.3	8.0	62.2	8.0	60.0	7.8
Sex (male: *n*, %)	757	61.1				
BMI (kg/m^2^: mean, SD)	25.8	4.5	26.1	4.4	25.2	4.7
With COPD (*n*, %)	999	80.6	618	81.6	381	79.0
Current smoker (*n*, %)	524	42.3	305	40.3	219	45.4
Pack‐years (mean, SD)	43.3	24.8	46.9	26.7	37.6	20.4
≥1 prevalent VF (*n*, %)	253	20.4	185	24.4	68	14.1
≥2 prevalent VF (*n*, %)	113	9.1	84	11.1	29	6.0
Grade 2 or 3 prevalent VF (*n*, %)	132	10.7	91	12.0	41	8.5
Kyphosis T_4_ to T_9_ (degrees: mean, SD)	26.4	7.7	25.8	7.8	27.5	7.6
Kyphosis T_4_ to T_12_ (degrees: mean, SD)	34.5	10.2	33.4	10.3	36.2	9.7
Incident VFs 1 year (*n*, %)	117	9.4	86	11.4	31	6.4
Incident VFs 3 years (*n*, %)	294	23.7	205	27.1	89	18.5

BMI = body mass index; COPD = chronic obstructive pulmonary disease; VF = vertebral fracture.

1 pack‐year = 20 cigarettes per day for 1 year.

Of all vertebrae that were evaluated (*n* = 12,063), 438 (3.6%) showed a VF grade 1 or higher at baseline (Table 2). Most VFs (63.0%) were located in the midthoracic (T_7_ to T_8_) and thoracolumbar area (T_11_ to T_12_; Supplemental Fig. S1).

**Table 2:** Number and Shape of Prevalent Vertebral Fractures Per Vertebral Level

	Vertebrae and VFs			VF by any deformation (overlapping)						VF by highest deformation (mutually exclusive)					
	Total no. scored	With VF		With (also) wedge shape		With (also) biconcave shape		With (also) biconcave shape		Wedge as highest deformation		Biconcave as highest deformation		Crush as highest deformation	
	*n*	*n*	%	*n*	% (of VF)	*n*	% (of VF)	*n*	% (of VF)	*n*	% (of VF)	*n*	% (of VF)	*n*	% (of VF)
T_4_	1236	14	1.1	7	50.0	11	78.6	2	14.3	3	21.4	10	71.4	1	7.1
T_5_	1233	24	1.9	19	79.2	16	66.7	4	16.7	14	58.3	8	33.3	2	8.3
T_6_	1227	38	3.1	34	89.5	18	47.4	1	2.6	29	76.3	9	23.7	0	0.0
T_7_	1232	74	6.0	72	97.3	26	35.1	1	1.4	65	87.8	9	12.2	0	0.0
T_8_	1235	85	6.9	81	95.3	26	30.6	2	2.4	74	87.1	11	12.9	0	0.0
T_9_	1236	43	3.5	36	83.7	21	48.8	4	9.3	28	65.1	13	30.2	2	4.7
T_10_	1236	13	1.1	12	92.3	6	46.2	0	0	9	69.2	4	30.8	0	0.0
T_11_	1231	50	4.1	43	86.0	21	42.0	2	4.0	39	78.0	10	20.0	1	2.0
T_12_	1198	67	5.6	59	88.1	27	40.3	3	4.5	49	73.1	18	26.9	0	0
L_1_	999	30	3.0	27	90.0	15	50.0	1	3.3	21	70.0	9	30.0	0	0
Total	12,063	438	3.6	390	89.0	187	42.7	20	4.6	331	75.6	101	23.1	6	1.4

VF = vertebral fracture.

Because of VF definitions by SpineAnalyzer morphometry software, VFs can have multiple configurations. In the section “VF by any deformation (overlapping)” any shape of VF was scored, and therefore VF shapes can overlap (for example: a vertebra with 41% biconcave and 24% wedge has two VF shapes). In the section “VF by highest deformation (mutually exclusive)” VFs were scored according to the highest deformation in percentage. These three columns are mutually exclusive.

Of all VFs, most VFs were wedge shaped (75.6%). An even larger proportion of VFs had height loss at the anterior side of the vertebral body (89.0%) but did not necessarily have wedge shape as largest deformation.

The mean kyphosis angle of subjects with one or more prevalent VF (*n* = 248) was 30.1 ± 9.3 degrees for angle T_4_ to T_9_, and 40.5 ± 10.6 for angle T_4_ to T_12_. Both kyphosis angles were significantly greater compared with subjects without prevalent VFs (*n* = 989, 25.5 ± 7.0 degrees for angle T_4_ to T_9_, and 33.0 ± 9.4 for angle T_4_ to T_12_, respectively).

After adjustment for age and sex, the mean kyphosis angle was significantly greater in subjects with multiple VFs (*n* = 108 with ≥2 VFs, mean T_4_ to T_9_ angle: 33.3 ± 10.0; mean T_4_ to T_12_ angle: 43.7 ±11.3) compared with subjects with only 1 VF (*p* < 0.001 for both angles) or without VFs (*p* < 0.001 for both angles). Also in subjects with severe VFs (*n* = 33 with at least 1 grade 3 VF, mean T_4_ to T_9_ angle: 36.2 ± 10.9; mean T_4_ to T_12_ angle: 46.1 ± 10.6), mean kyphosis angle was significantly greater compared with subjects with a grade 2 VF (*p* < 0.001 for T_4_ to T_9_ angle; *p* = 0.003 for T_4_ to T_12_ angle), subjects with a grade 1 VF (*p* < 0.001 for T_4_ to T_9_ angle; *p* = 0.006 for T_4_ to T_12_ angle), or subjects without VFs (*p* < 0.001 for both angles). The same applied to subjects with an SDI of ≥5 (*n* = 36, mean T_4_ to T_9_ angle: 37.1 ±11.5; mean T_4_ to T_12_ angle: 48.2 ±12.6) compared with subjects with an SDI of 3 to 4 (*p* = 0.002 for T_4_ to T_9_ angle; *p* = 0.004 for T_4_ to T_12_ angle), with an SDI of 1 to 2 (*p* < 0.001 for both angles), or subjects without VFs (*p* < 0.001 for both angles).

In line with prevalent VFs, most incident VFs occurred in T_7_ to T_8_ and T_11_ to T_12_ (56% and 58% for 1‐year and 3‐year incidence). For the 1‐year incidence, also T_6_ (13%) was a frequent location for incident VFs.

The mean increase of the kyphosis angle in the total population within 1 (ΔT_4_ to T_9_: 0.3 ± 2.6; ΔT_4_ to T_12_: 0.3 ± 3.7) and within 3 years (ΔT_4_ to T_9_: 1.2 ± 2.8; ΔT_4_ to T_12_: 1.4 ± 4.0) was small. The mean increase was larger in subjects with incident VFs compared with subjects without incident VFs (Table 3).

**Table 3:** Change in Kyphosis Angles Within 1 and 3 Years in Subjects With or Without Incident Vertebral Fractures

	One‐year incidence						Three‐year incidence					
	Without incident VF *n* = 1117		With incident VF *n* = 117		*p* Value		Without incident VF *n* = 943		With incident VF *n* = 294		*p* Value	
	Mean	SD	Mean	SD	*	**	Mean	SD	Mean	SD	*	**
Kyphosis T_4_ to T_9_	25.9	7.4	31.1	9.3	<0.0001		25.4	7.1	29.7	8.9	<0.0001	
Kyphosis T_4_ to T_12_	33.8	9.8	40.7	10.8	<0.0001		32.9	9.5	39.6	10.6	<0.0001	
ΔT_4_ to T_9_ within 1 year	0.3	2.5	0.8	2.9	0.0277	0.0040						
ΔT_4_ to T_12_ within 1 year	0.2	3.6	1.3	4.2	0.0045	0.0000						
ΔT_4_ to T_9_ within 3 years							1.0	2.7	1.7	3.2	0.0003	0.0004
ΔT_4_ to T_12_ within 3 years							1.2	3.9	2.2	4.1	0.0002	<0.0001

VF = vertebral fracture.

*Adjusted for age and sex.

**Adjusted for age, sex, and kyphosis at baseline

All kyphosis angles and change in kyphosis angles are given in degrees.

In subjects with an increase in SDI of >2 within 3 years, the increase in kyphosis angle (*n* = 77, ΔT_4_ to T_9_ angle: 3.0 ± 3.8 and ΔT_4_ to T_12_ angle: 4.4 ± 4.3) was significantly higher than in subjects without incident VFs, with an increase in SDI of 1 or 2 (*p* < 0.01 for both angles). The increase in kyphosis in subjects with an increase in SDI of >2 within 1 year (*n* = 10) was not significantly different from the other groups.

In univariate models, both T_4_ to T_9_ and T_4_ to T_12_ kyphosis angles at baseline were significantly associated with incident VFs within 1 and within 3 years (Table 4). In multivariate models, the baseline kyphosis angle remained a significant determinant of incident VFs. However, a prevalent VF was a much stronger determinant.

**Table 4:** Univariate and Multivariate Associations Between Baseline Kyphosis Angle and Risk of Incident Vertebral Fractures Within 1 and 3 Years

	Univariate		Multivariate with T_4_ to T_9_		Multivariate with T_4_ to T_12_	
	HR	95% CI	HR	95% CI	HR	95% CI
One‐year incidence						
Age (per +8 years)	1.42	(1.167–1.738)	0.99	(0.789–1.251)	0.97	(0.771–1.225)
Sex (male versus female)	1.78	(1.179–2.679)	1.65	(1.082–2.525)	1.83	(1.182–2.841)
BMI (per +5 kg/m^2^)	0.85	(0.696–1.049)	–		–	
Pack‐years (per +25 pack‐years)	1.06	(0.895–1.264)	–		–	
Smoking status (current versus former smoker)	0.79	(0.542–1.150)	–		–	
With COPD (versus no COPD)	1.64	(0.955–2.823)	0.88	(0.492–1.587)	0.83	(0.444–1.538)
GOLD stage II (versus no COPD)	1.45	(0.803–2.611)	–		–	
GOLD stage III (versus no COPD)	1.57	(0.867–2.828)	–		–	
GOLD stage IV (versus no COPD)	2.74	(1.392–5.390)	–		–	
≥1 prevalent VF (versus no VF)	5.41	(3.749–7.799)	3.30	(2.181–4.987)	3.20	(2.096–4.898)
BA (per –47 HU)	2.00	(1.618–2.475)	1.39	(1.104–1.761)	1.46	(1.147–1.856)
Kyphosis T_4_ to T_9_ (per +8 degrees)	1.70	(1.453–1.978)	1.31	(1.113–1.533)	–	
Kyphosis T_4_ to T_12_ (per +10 degrees)	1.76	(1.489–2.076)	–		1.34	(1.121–1.608)
Three‐year incidence						
Age (per +8 years)	1.30	(1.154–1.473)	1.03	(0.892–1.184)	1.01	(0.872–1.162)
Sex (male versus female)	1.47	(1.147–1.886)	1.33	(1.029–1.717)	1.41	(1.082–1.828)
BMI (per +5 kg/m^2^)	0.93	(0.815–1.053)	–		–	
Pack‐years (per +25 pack‐years)	1.10	(0.990–1.222)	–		–	
Smoking status (current versus former smoker)	0.93	(0.736–1.172)	–		–	
With COPD (versus no COPD)	1.30	(0.947–1.777)	1.03	(0.729–1.445)	1.00	(0.699–1.433)
GOLD stage II (versus no COPD)	1.22	(0.863–1.719)	–		–	
GOLD stage III (versus no COPD)	1.32	(0.934–1.865)	–		–	
GOLD stage IV (versus no COPD)	1.55	(0.992–2.426)	–		–	
≥1 prevalent VF (versus no VF)	3.88	(3.087–4.873)	2.82	(2.178–3.644)	2.62	(2.006–3.413)
BA (per –47 HU)	1.60	(1.410–1.822)	1.23	(1.068–1.413)	1.26	(1.086–1.450)
Kyphosis T_4_ to T_9_ (per +8 degrees)	1.47	(1.324–1.628)	1.21	(1.068–1.344)	–	
Kyphosis T_4_ to T_12_ (per +10 degrees)	1.58	(1.420–1.757)	–		1.29	(1.147–1.448)

VF = vertebral fracture; HR = hazard ratio; CI = confidence interval; BMI = body mass index; COPD = chronic obstructive pulmonary disease; GOLD = Global Initiative for Chronic Obstructive Lung Disease.

For continuous variables, HRs are given per standard deviation.

## Discussion

In this study, we showed that prevalent VFs are associated with greater kyphosis angles and that greater kyphosis angles at baseline are independently associated with incident VFs, within 1 and 3 years. Although a prevalent VF is a stronger determinant, both baseline BA and kyphosis angle contribute to incident VF risk. Our data support the theory that greater kyphosis angle contributes to higher biomechanical loads in the spine and hence may lead to increased VF risk.

In line with literature,^39^, ^40^, ^41^ we found that both prevalent and incident VFs were observed most frequently in T_7_ to T_8_ and T_11_ to T_12_. A computational model of the spine showed that during daily activities, vertebral compressive load was highest in the thoracolumbar (T_11_ to L_1_) and lumbar spine (L_2_ to L_5_). Because of the higher vertebral strength in the lumbar spine, the risk of VFs was highest in the thoracolumbar area and in vertebra T_6_ during some activities.^27^ These findings could explain the high prevalence and incidence of VFs in the thoracolumbar area.

Similar to previous results,^38^ we found significant associations between prevalent VFs and baseline kyphosis angle and between incident VFs and increase in kyphosis angle after 1‐ and 3‐year follow‐up.

Our data also showed that a greater baseline kyphosis angle was an independent determinant for incident VFs. Bruno and colleagues showed in a computational model that with greater kyphosis angles, the load within the spine was higher during daily activities than in less kyphotic spinal models.^28^ In line with our data, Roux and colleagues also found an independent association between kyphosis angle and incident VFs,^29^ but Katzman and colleagues did not.^30^ Different methods of kyphosis measurement, imaging methods, and positioning of patients were used (measured on left lateral decubitus position X‐ray (T_4_ to T_12_ angle); Debrunner kyphometer in standing position (C_7_ to T_12_ angle), whereas we used CT scans taken in supine position. In addition, patient populations were slightly different (both studies included women only, selected based on prevalence of VFs, or based on *T*‐score, and the population in the study by Roux and colleagues was older).

The associations we found support the hypothesis that the load‐to‐bone strength ratio is highest in the thoracolumbar area and during some activities in the high/midthoracic area and that the biomechanical effect of greater kyphosis angle could contribute to a higher load‐to‐bone strength ratio.

This study has some limitations. First, there is selection bias; only former and current smokers of non‐Hispanic white ethnicity with or without COPD were included. Subjects were recruited from outpatient clinics with GOLD II, GOLD III, or GOLD IV (with COPD) or through site databases and advertisement (without COPD). Subjects using oral GC at baseline were excluded, and there was no information available about history of steroid use or use of medications such as bisphosphonates. We excluded subjects with incomplete set of CT scans of insufficient quality. In addition, only a limited number of GOLD IV subjects (*n* = 111) and subjects without COPD (*n* = 240) were included. These inclusion and exclusion criteria limit the applicability to the general COPD and/or (former) smoker population.

Second, kyphosis angles were measured on chest CT images taken in supine position. It is expected that in standing position, gravitational forces influence thoracic kyphosis to a higher extent than in supine position, leading to an underestimation of the measured kyphosis angle in our study. However, studies comparing kyphosis angles in supine and in standing position showed that these measures are well associated.^42^, ^43^ Therefore, supine images could serve as an alternative to standing recordings for kyphosis measurement.

In addition, only the thoracic spine and first lumbar vertebrae were imaged on chest CT, and therefore prevalent and incident VFs in the lumbar spine, as well as lordosis angles could not be measured.

Furthermore, kyphosis angles were measured for this research using a new method, via a third‐order polynomial fit through user‐indicated points in the spine. Using this method, the measured angles describe the curvature of the spine rather than the influence of individual endplate deviations. The method is depending on user input, but precision in repeated measures, as well as correlations with another angle measurement method were very good.

Lastly, BA is not a standardized method of bone density measurement. However, there are several studies showing the associations between BA by CT and BMD by DXA, or between BA by CT and vertebral fractures.^44^

In this study, we found an association between prevalent VFs and CT‐measured baseline kyphosis angle, and between incident VFs and increase in kyphosis angle. In addition, baseline kyphosis angle was associated with short‐term VF incidence after adjustment for BA and prevalent VFs. These results support the theory that greater kyphosis angles contribute to higher biomechanical loads in the spine and may attribute to short‐term VF risk.

## Disclosures

PG reports grants, speaker fees, and advisory board from Amgen, grants from Pfizer, grants from MSD, grants from UCB, grants from Abbott, grants and speaker fees from Lilly, grants from BMS, grants from Novartis, grants from Roche, and grants from Will Pharma, outside the submitted work. EFMW reports board membership at Boehringer, grants and speaker fees from AstraZeneca, grants and speaker fees from GSK, speaker fees from Novartis, and speaker fees from Chiesi, outside the submitted work. JPWvdB reports grants from Eli Lilly, grants from Will Pharma, and grants from Amgen, outside the submitted work. All other authors state that they have no conflicts of interest.

## Supporting information

Supporting Figure S1.
