# Supplementary material for: Thoracic Kyphosis on Chest CT Scans Is Associated With Incident Vertebral Fractures in Smokers
Source: J Bone Miner Res. 2019 Feb 27;34(5):e3672. doi: 10.1002/jbmr.3672 (PMC6946934; doi:10.1002/jbmr.3672)
Supplement: Supplementary file 1 — Supporting Figure S1. [file JBMR-34-859-s001.docx]

**Online supplement**


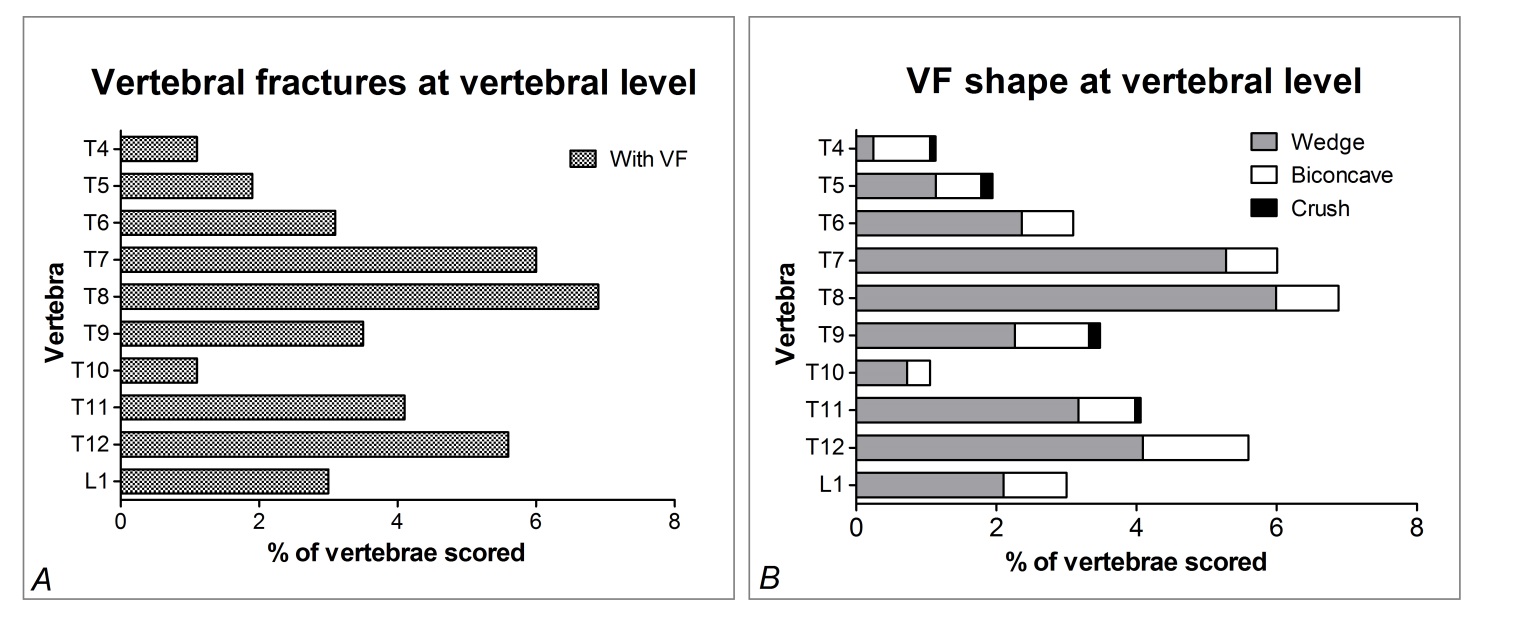


**e-Figure 1** Number and shape of prevalent vertebral fractures

*A)* Percentage of vertebrae with vertebral fractures (VFs) of any shape, and *B)* Shapes of vertebral fractures

VFs are expressed in % of the vertebrae that were scored at that specific vertebral level. The number of vertebrae scored at each level can be found in Table 2.
